# Topical Fluticasone Propionate Versus Fusidic Acid for Zoon’s Balanitis in Men: A Prospective, Open-Label, Non-Randomised Comparative Study with an Exploratory Long-Term Assessment

**DOI:** 10.3390/jcm15218515

**Published:** 2026-11-02

**Authors:** Katarzyna Czajkowska, Mateusz Czajkowski, Roman Janusz Nowicki, Marcin Matuszewski, Małgorzata Sokołowska-Wojdyło

**Affiliations:** 1 Department of Dermatology, Venerology and Allergology, Medical University of Gdańsk, Mariana Smoluchowskiego 17, 80-214 Gdańsk, Poland; roman.nowicki@gumed.edu.pl (R.J.N.); malgorzata.sokolowska-wojdylo@gumed.edu.pl (M.S.-W.); 2 Department of Urology, Medical University of Gdańsk, Mariana Smoluchowskiego 17, 80-214 Gdańsk, Poland; mateusz.czajkowski@gumed.edu.pl (M.C.); marcin.matuszewski@gumed.edu.pl (M.M.)

**Keywords:** plasma cell balanitis, Zoon balanitis, topical therapy, fluticasone propionate, fusidic acid

## Abstract

**Background**: Prospective comparative evidence for topical treatment of Zoon’s balanitis is limited. We evaluated early clinical changes during two topical regimens and explored recorded long-term outcomes. **Methods**: In this single-centre, prospective, open-label, non-randomised study, 37 men with histopathologically confirmed Zoon’s balanitis were allocated by alternation to fluticasone propionate 0.05% (*n* = 18) or fusidic acid 2% (*n* = 19) for four weeks. Early assessments used a four-point study Investigator’s Global Assessment (IGA) and symptom visual analogue scales. Additional exploratory comparisons included IGA 0 at week 4. A separate categorical assessment was undertaken in 2026. The study was not registered in a public clinical-trial registry. **Results**: Mean IGA decreased from 2.17 to 0.67 with fluticasone and from 2.26 to 0.89 with fusidic acid between baseline and week 4. Complete clearance occurred in 8/18 (44.4%) and 6/19 (31.6%) participants, respectively; the absolute difference was 12.9 percentage points (95% confidence interval, −17.1 to 40.1; Fisher’s exact *p* = 0.50768). Among participants assessable in 2026, recorded remission occurred in 12/16 (75.0%) and 3/15 (20.0%), respectively (unadjusted odds ratio, 12.00; approximate 95% confidence interval, 2.20–65.52; *p* = 0.00385). These data did not establish uninterrupted or treatment-free remission; missing-outcome sensitivity analyses limited the robustness of the association. **Conclusions**: Clinical severity improved during both regimens, but comparative equivalence was not established. The association between initial fluticasone allocation and recorded remission in 2026 is hypothesis-generating and requires confirmation in a registered, adequately powered controlled study with standardised long-term assessment.

## 1. Introduction

Zoon’s balanitis (ZB), also known as plasma cell balanitis or balanitis circumscripta plasmacellularis, is a benign chronic inflammatory dermatosis that primarily affects the glans penis and, less frequently, the inner prepuce. It usually occurs in older uncircumcised men and presents as a well-demarcated, shiny erythematous or reddish-brown patch or plaque, frequently with “cayenne pepper” speckling caused by microhaemorrhage and haemosiderin deposition [1,2,3]. Its true frequency is probably underestimated because mild or minimally symptomatic disease may not prompt medical evaluation. In published cohorts of men assessed for inflammatory penile dermatoses, ZB accounted for 8–27.8% of diagnoses [4,5,6]. Paediatric cases are uncommon [7,8], while reports of cases in circumcised men have mainly involved immunodeficiency or incomplete circumcision [2,9].

The pathogenesis of this disease remains incompletely understood. The strong association between an intact foreskin and high remission rates after circumcision supports the roles of chronic occlusion, friction, moisture, irritant exposure, and retention of urine or smegma [3,10,11]. Mechanical irritation, heat, repetitive trauma, and concomitant infections may contribute to disease exacerbation [1,12]. Although infectious triggers have been proposed, the available evidence does not support a primarily infection-driven mechanism or a consistent association with human papillomavirus [1,12,13]. ZB may clinically mimic penile intraepithelial neoplasia; therefore, histopathological confirmation and clinicopathological correlation are important in atypical, uncertain, or treatment-resistant lesions [14,15,16]. Recent evidence has also questioned whether ZB is always a distinct entity, suggesting that “zoonoid” plasmacytic inflammation may be a reaction pattern accompanying other penile dermatoses, particularly in male genital lichen sclerosus [17]. Molecular pathways remain poorly characterised; a small study found no difference in NFKB1 or NFKB2 mRNA expression between ZB and control samples [18].

The European guideline lists circumcision (1,C), topical corticosteroids with or without an antibacterial agent (2,D), and antibacterial cream monotherapy such as mupirocin (2,D) among management options for Zoon’s balanitis; fusidic acid itself is not specifically listed [19]. Topical management nevertheless remains relevant when surgery is declined or deferred. Published conservative-treatment evidence consists largely of small series and reports [1,12]. Fluticasone propionate is a topical corticosteroid with established dermatological use [20,21], whereas fusidic acid monotherapy has been reported as a potential alternative approach [22]. Proposed anti-inflammatory properties offer a rationale for investigation but do not establish clinical efficacy in this condition [23,24]. Our comparison examined responses during two single-agent regimens; a corticosteroid–antibacterial combination would address a different treatment question. We therefore evaluated early physician-rated and patient-reported outcomes and an exploratory assessment in 2026, without seeking to establish equivalence or to compare monotherapy with combination treatment.

## 2. Materials and Methods

### 2.1. Study Design and Setting

This was a single-centre, prospective, open-label, non-randomised study with alternating allocation conducted at the Department of Dermatology, Venereology and Allergology, Medical University of Gdańsk. Recruitment, treatment and early follow-up were conducted during 2017–2022. An additional categorical outcome assessment was undertaken in 2026 as an exploratory extension. The interval to this assessment was not assumed to be identical for all participants. The original study protocol included prospective clinical assessments during the four-week treatment period and a follow-up visit three months after treatment discontinuation. The additional long-term assessment conducted in 2026 was not planned at study initiation and was undertaken as a subsequent exploratory evaluation. The Bioethics Committee for Scientific Research at the Medical University of Gdańsk approved the study (NKBBN/249/2017; 3 July 2017), and all participants provided written informed consent. The study was not registered in a public clinical-trial registry, and no registration number is available.

### 2.2. Participants and Diagnostic Confirmation

Eligible participants were men aged 18 years or older with clinically suspected Zoon’s balanitis supported by baseline dermoscopic findings and confirmed by histopathological examination of a penile-lesion biopsy. Biopsy specimens were obtained using a 4-mm punch. Histopathological assessment was performed by an experienced dermatopathologist based on characteristic morphological features and clinicopathological correlation. Exclusion criteria were histopathological evidence of premalignancy or malignancy, an alternative diagnosis requiring different management, or clinically active bacterial, viral or fungal balanitis requiring specific treatment. These exclusions were established through clinical examination and histopathological assessment of biopsy specimens. Alternative diagnoses, including lichen sclerosus and penile intraepithelial neoplasia, were excluded by clinicopathological correlation and the absence of characteristic histopathological features.

### 2.3. Allocation and Interventions

Participants were assigned in an approximately 1:1 ratio using an alternating sequence to fluticasone propionate 0.05% cream (0.5 mg/g; Cutivate^®^, GlaxoSmithKline, London, UK) once daily or fusidic acid 2% cream (20 mg/g; Fucidin^®^, LEO Pharma A/S, Ballerup, Denmark) twice daily for four weeks. Allocation was implemented by K.C. using an alternating sequence, with the first participant assigned to the fluticasone propionate group and each subsequent participant assigned alternately to the fusidic acid and fluticasone propionate groups. This was not random allocation, and there was no concealed randomisation; the alternating assignment was predictable.

Participants applied a thin layer to all visible lesions after gentle cleansing with water and careful drying. Occlusive dressings were not used. Other topical corticosteroids, calcineurin inhibitors, antibiotics, antifungals, antiseptics and procedural treatments for Zoon’s balanitis were prohibited during the initial treatment period. Adherence was assessed at visits by K.C.; however, quantitative data on missed doses were not systematically recorded. No participants discontinued the 4-week treatment, and quantitative data on protocol deviations were not systematically collected. After the initial four-week treatment period until the end of 3 months follow-up, patients in both groups were instructed to refrain from using topical corticosteroids, calcineurin inhibitors, antibiotics, antifungals, antiseptics, or procedural treatments for Zoon’s balanitis, consistent with the restrictions applied during the initial treatment period.

### 2.4. Assessments and Outcomes

Assessments were scheduled at baseline, weeks 2 and 4, and three months after treatment discontinuation. The same investigator, K.C., performed the early clinical assessments and was aware of treatment allocation. Clinical photographs and dermoscopic images were obtained using a FotoFinder system with a Medicam 800 HD camera (FotoFinder Systems GmbH, Bad Birnbach, Germany). At each study visit, the treated area was clinically examined for local adverse effects, including skin atrophy, telangiectasia, and signs of secondary infection.

Lesion severity was graded using a four-point study IGA based on the clinical severity assessment described by Kyriakou et al. [25], where it was referred to as a four-point Likert scale: 0, no lesions; 1, mild lesions with slight erythema; 2, moderate lesions with erythema and petechiae; and 3, severe lesions with intense erythema, petechiae, and erosions. This four-point scale was adapted from the previously published clinical severity assessment by Kyriakou et al. [25] and has not been formally validated as an outcome measure specifically for Zoon’s balanitis. Pain, burning, itch and dyspareunia were assessed on a 0–10 visual analogue scale (VAS). Entries indicating no sexual activity were treated as not applicable to dyspareunia rather than as a zero score. Adverse-event assessment consisted of direct questioning about treatment-related symptoms, including local burning or irritation, and clinical examination of the treated area at each follow-up visit for signs of local adverse reactions.

The originally stated primary short-term outcome was within-group change in IGA across the scheduled assessments. Between-group comparisons at individual visits and changes in symptoms were secondary analyses. Additional exploratory comparisons assessed change from baseline to week 4 and to three months, complete clearance (IGA 0), clear or mild disease (IGA 0–1), and improvement of at least one IGA grade from baseline. These additional endpoints were not retrospectively designated as prespecified primary outcomes.

The 2026 assessment was undertaken by K.C. during an in-person clinical follow-up visit. Remission was defined as the absence of clinically detectable lesions of Zoon’s balanitis on physical examination. The available long-term dataset records remission, persistent/recurrent disease without remission, subsequent circumcision, or non-assessable status, with death and loss to follow-up distinguished where recorded. There were no standardised numerical IGA or VAS measurements in this dataset for 2026. We therefore report “recorded remission at the 2026 assessment”, not uninterrupted sustained remission or remission attributable solely to the initial course. Circumcision was analysed as a separate subsequent-management category.

### 2.5. Post-Treatment Management

During the three-month post-treatment follow-up period, patients in both groups were instructed to refrain from using topical corticosteroids, calcineurin inhibitors, antibiotics, antifungals, antiseptics, or procedural treatments for Zoon’s balanitis. The categorical long-term dataset alone does not establish the absence of subsequent topical or other treatment. Circumcision was not automatically equated with failure of the assigned topical regimen.

### 2.6. Sample Size

The analysis included the 37 enrolled participants. No formal a priori sample-size calculation was performed. Interpretation therefore focuses on effect estimates and confidence intervals rather than a claim that the cohort had sufficient power to establish equivalence or detect modest treatment differences.

### 2.7. Statistical Analysis

Analyses used available numeric values without replacing missing or non-applicable entries with zero. Denominators are reported for each outcome and time point. Longitudinal Friedman tests used participants with numeric measurements at all four early assessments for the outcome concerned. All statistical analyses were exploratory. No adjustment for multiple comparisons was applied; therefore, reported *p* values are nominal and should be interpreted with caution. A separate, revision-stage Holm adjustment was performed for the seven evaluable symptom-by-group Friedman tests, as described below.

Ordinal IGA and VAS outcomes were summarised descriptively. Between-group comparisons of scores and change scores used two-sided Mann–Whitney U tests with asymptotic tie correction and without continuity correction. Within-group trajectories used the Friedman test with its asymptotic chi-square reference distribution. These omnibus tests were not interpreted as individual baseline-to-week-4 pairwise tests. No pain-trajectory test was estimated for the fluticasone group because every value was zero.

Binary exploratory outcomes were compared using two-sided Fisher’s exact tests. Proportion confidence intervals used the Wilson method; confidence intervals for differences between independent proportions used Newcombe’s hybrid-score method [26]. Unadjusted odds ratios are reported with approximate log-Wald 95% confidence intervals. No equivalence or non-inferiority margin was specified.

Long-term categories are presented using the original group denominators. The comparison of recorded remission among assessable participants is reported alongside the full-cohort proportion with documented remission; the latter is not a claim that non-assessable participants had clinical failure. Sensitivity analyses include mathematical extreme assignments for all six unassessable outcomes and separate analyses excluding the four deaths while varying only the two remaining losses to follow-up. The all-six bounds are not imputations of living remission status after death. Analyses excluding deaths describe a survivor subset.

Secondary, revision-stage and long-term comparisons are exploratory, with nominal *p* values reported descriptively. As an additional revision-stage sensitivity analysis, Holm adjustment was applied across the seven evaluable symptom-by-group Friedman tests. This adjustment does not provide family-wise error control across all analyses in the manuscript. The original clinical analyses were reported using IBM SPSS Statistics version 23.0 (IBM Corp., Armonk, NY, USA); the source-data reanalysis for this revision was performed using Python (version 3.11; Python Software Foundation, Wilmington, DE, USA) and SciPy (version 1.17.0; https://scipy.org/).

## 3. Results

### 3.1. Participants and Baseline Characteristics

Thirty-seven men were included: 18 received fluticasone propionate and 19 received fusidic acid. Mean age was 65.86 years (range, 29–84), mean body mass index was 27.67 kg/m^2^, and mean symptom duration before diagnosis was 24 months (range, 1–84). Baseline demographic and clinical characteristics, including type 2 diabetes mellitus, immunosuppression, phimosis, lesion location, baseline IGA category, and smoking status, are presented in Table 1.

**Figure 1:**
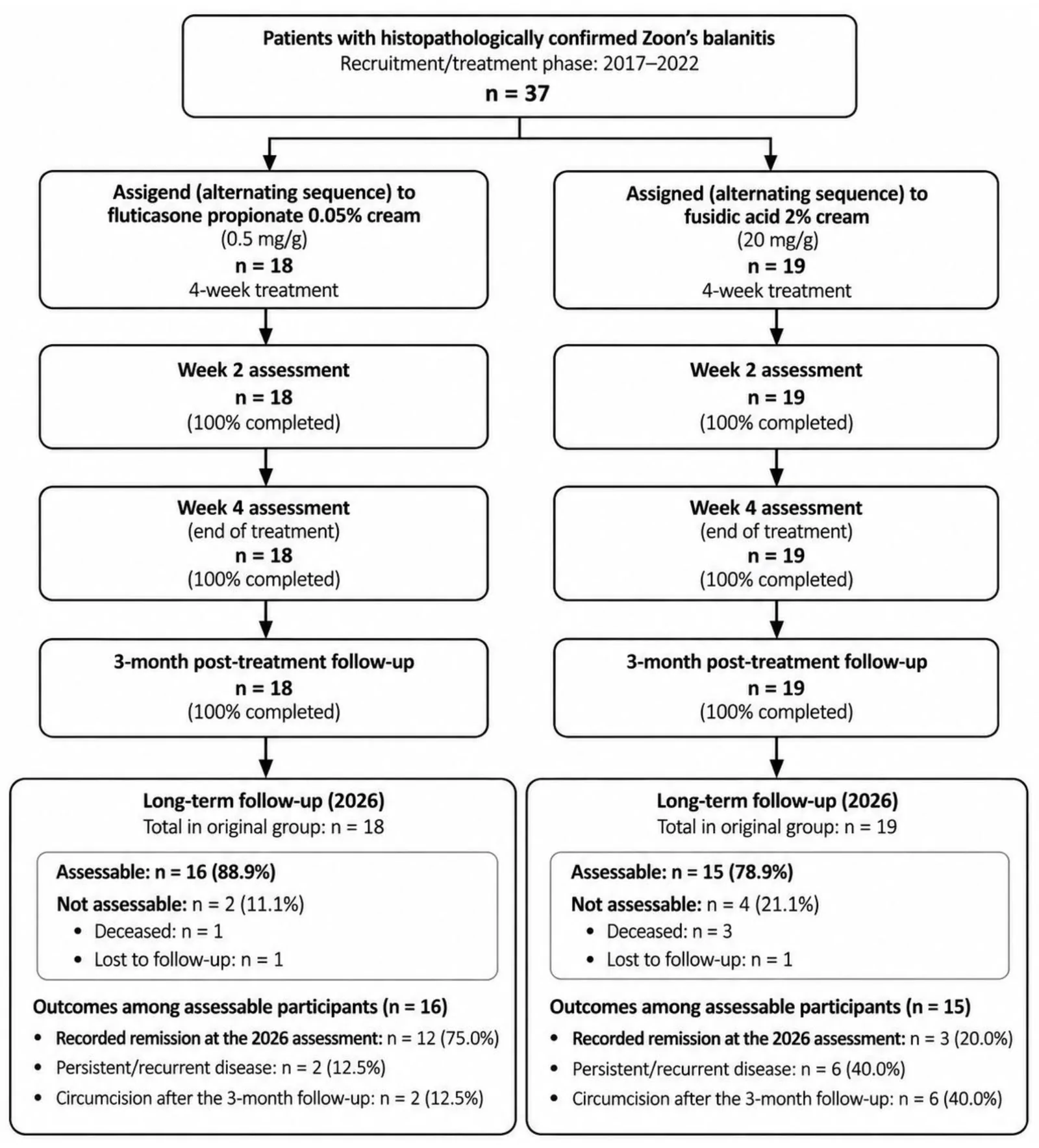
Study Flow-chart.

Numeric IGA measurements were available for all 37 participants at baseline, week 2, week 4, and 3 months after treatment discontinuation (18/18 in the fluticasone group and 19/19 in the fusidic acid group at each assessment). Availability of these measurements was considered separately from treatment adherence and protocol deviations (Figure 1).

### 3.2. Clinical Severity

**Figure 2:**
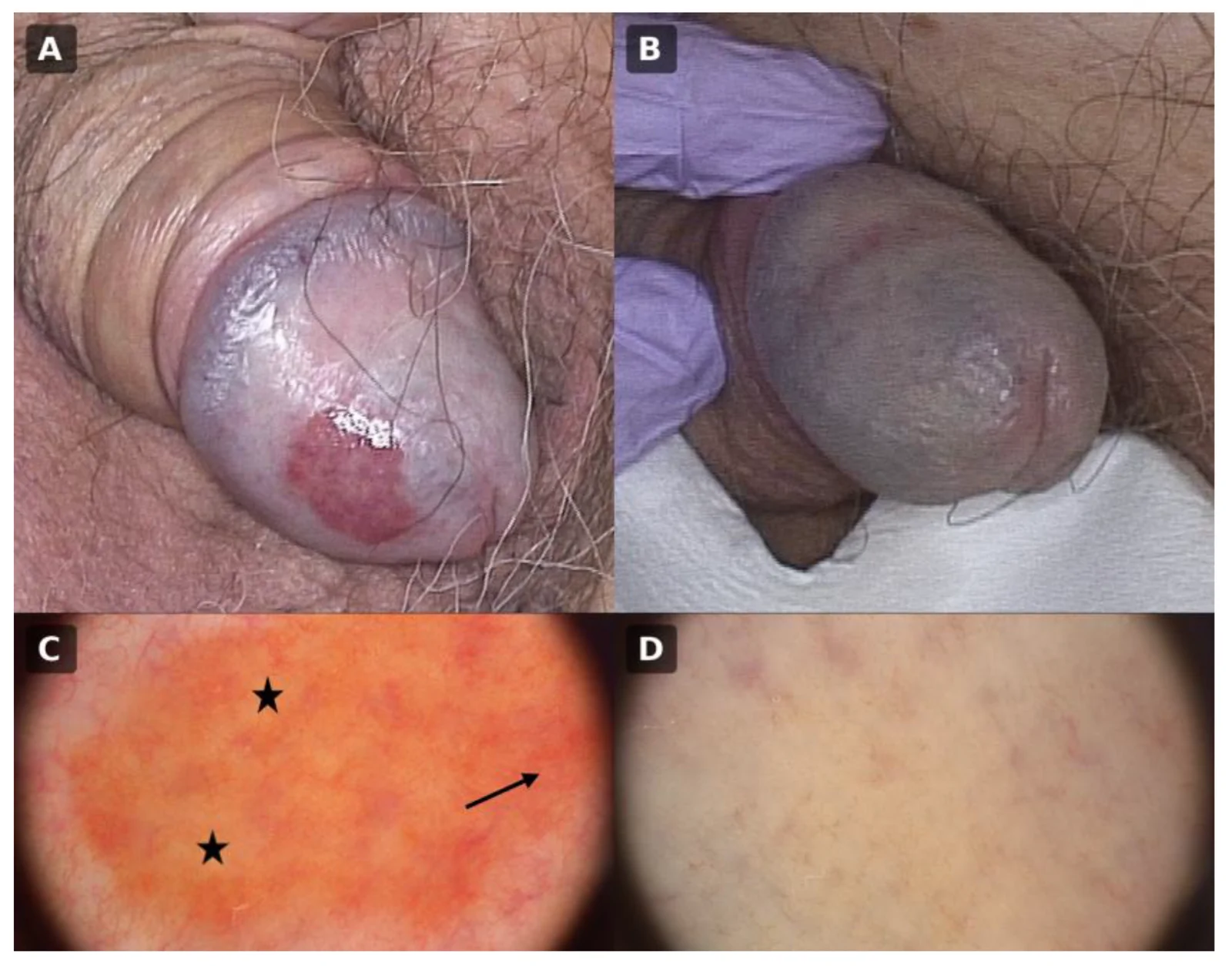
Representative clinical and dermoscopic images of Zoon’s balanitis before and after treatment. (**A**) Clinical appearance at baseline (IGA score: 2); (**B**) clinical appearance after treatment (IGA score: 0); (**C**) dermoscopic appearance at baseline, showing orange-yellow structureless areas (asterisk) and curved vessels (arrow); and (**D**) dermoscopic appearance after treatment, with no characteristic dermoscopic features remaining.

IGA scores decreased during treatment in both groups. In the fluticasone group (*n* = 18), mean IGA scores were 2.17 at baseline, 1.11 at week 2, 0.67 at week 4, and 1.00 at three months after treatment discontinuation. Corresponding mean scores in the fusidic acid group (*n* = 19) were 2.26, 1.05, 0.89, and 1.11, respectively. IGA measurements were available for all participants at all four scheduled assessments (Figure 2).

The distributions across IGA categories 0/1/2/3 were 0/3/9/6, 2/12/4/0, 8/8/2/0, and 5/8/5/0 in the fluticasone group and 0/1/12/6, 3/13/2/1, 6/9/4/0, and 5/8/5/1 in the fusidic acid group at baseline, week 2, week 4, and three months, respectively (Table 2).

At week 4, complete clearance (IGA 0) was observed in 8/18 (44.4%) participants in the fluticasone group and 6/19 (31.6%) in the fusidic acid group. Clear or mild disease (IGA 0–1) was observed in 16/18 (88.9%) and 15/19 (78.9%) participants, respectively. At three months, complete clearance was observed in 5/18 (27.8%) and 5/19 (26.3%) participants, respectively. Additional exploratory response estimates are reported in Table S1. The absence of statistically significant between-group differences should not be interpreted as evidence of equivalence.

### 3.3. Patient-Reported Symptoms

Patient-reported symptom burden was generally low. Itch decreased significantly over time in both groups. In the fluticasone group, the mean itch score decreased from 0.67 at baseline to 0 at week 2 and remained 0 through the 3-month assessment (Friedman *p* = 0.029). In the fusidic acid group, the mean itch score decreased from 1.16 at baseline to 0.42 at week 2, 0.32 at week 4, and 0.21 at 3 months (Friedman *p* = 0.009). Burning decreased significantly over time in the fluticasone group (Friedman *p* = 0.012), reaching 0 at week 4 and remaining 0 at 3 months, whereas no statistically significant change was observed in the fusidic acid group (Friedman *p* = 0.080). Pain was absent at all assessments in the fluticasone group. In the fusidic acid group, mean pain decreased from 1.63 at baseline to 0.21 at weeks 2 and 4 and 0.16 at 3 months (Friedman *p* < 0.001). The between-group difference in pain was significant at baseline (*p* = 0.005), but not at subsequent assessments. Dyspareunia was analysed only in participants with evaluable numeric responses (14/18 in the fluticasone group and 16/19 in the fusidic acid group); participants with documented absence of sexual activity were classified as not applicable. Dyspareunia did not change significantly over time in either the fluticasone (Friedman *p* = 0.392) or fusidic acid group (Friedman *p* = 0.069) (Table 3).

### 3.4. Safety

Two adverse events were recorded. One participant in the fluticasone group (1/18, 5.6%) was identified with condylomata acuminata during follow-up, which were successfully treated with cryotherapy; the procedure was directed at the condylomata and not at Zoon’s balanitis. One participant in the fusidic acid group (1/19, 5.3%) reported mild application-site burning early during treatment, which did not require interruption or discontinuation of the assigned treatment. No formal causality assessment was recorded. No additional adverse events were recorded during the treatment course and early follow-up. Safety findings were limited to adverse events recorded during the treatment course and early follow-up.

### 3.5. Exploratory Assessment in 2026

Long-term status was assessable for 31/37 participants (83.8%) in 2026: 16/18 in the fluticasone group and 15/19 in the fusidic acid group. The six non-assessable participants comprised one death and one loss to follow-up in the fluticasone group and three deaths and one loss to follow-up in the fusidic acid group.

Follow-up duration was calculated from completion of the initial 4-week treatment to the individual long-term assessment in February 2026. Among the 31 assessable participants, the median follow-up duration was 79.5 months (IQR, 72.9–95.8; range, 44.6–103.4). The corresponding median durations were 79.4 months (IQR, 72.9–94.6; range, 44.6–103.1) in the fluticasone group and 79.5 months (IQR, 73.0–96.9; range, 44.8–103.4) in the fusidic acid group.

Using the full enrolled denominators, documented remission at the 2026 assessment was recorded in 12/18 (66.7%) participants originally assigned to fluticasone and 3/19 (15.8%) assigned to fusidic acid (unadjusted odds ratio [OR], 10.67; approximate 95% CI, 2.21–51.53; Fisher’s exact *p* = 0.00255). These proportions describe documented remission and do not classify participants with unavailable long-term outcomes as observed treatment failures. Among participants with assessable long-term status, remission at the 2026 assessment was recorded in 12/16 (75.0%) and 3/15 (20.0%), respectively (unadjusted OR, 12.00; approximate 95% CI, 2.20–65.52; Fisher’s exact *p* = 0.00385), corresponding to an absolute difference of 55.0 percentage points (95% CI, 19.9–74.7).

Persistent or recurrent disease without documented remission was recorded in two fluticasone-treated and six fusidic acid-treated participants. Eight participants underwent circumcision after completion of the 3-month follow-up period: two in the fluticasone group and six in the fusidic acid group. Four circumcisions were performed because of worsening or recurrent Zoon’s balanitis, with clinically active disease before surgery, whereas four were performed according to patient preference rather than because of disease worsening. None was performed because of phimosis (Table 4). Sensitivity analyses are presented in Table S2.

Under the adverse mathematical assignment across all six unassessable outcomes, remission was assigned to 12/18 participants in the fluticasone group and 7/19 in the fusidic acid group (Fisher’s exact *p* = 0.10314); thus, the association did not remain statistically significant under every possible missing-outcome assignment. When deaths were excluded and only the two living participants lost to follow-up were varied, the corresponding adverse scenario was 12/17 versus 4/16 (*p* = 0.01492). The former analysis represents a mathematical bound rather than an imputation of remission status after death, whereas the latter is restricted to a survivor subset. Because the 2026 assessment was categorical and did not provide continuous longitudinal ascertainment throughout the intervening period, these findings should not be interpreted as evidence of uninterrupted remission or as establishing a causal long-term durability advantage of the initial treatment regimen.

## 4. Discussion

In this prospective, open-label, non-randomised comparison, clinical severity decreased during four weeks of topical fluticasone propionate or fusidic acid. The early data did not establish a statistically significant between-group difference, but neither the design nor the precision of the estimates supports equivalence. In particular, the week-4 complete-clearance difference had a confidence interval compatible with benefit in either direction. Because there was no untreated or vehicle control, within-group improvement alone cannot separate treatment effects from the natural course of disease or other common components of care.

The exploratory 2026 findings should be interpreted differently from the early assessments. Documented recorded remission at the 2026 assessment was more frequent among participants initially assigned to fluticasone, but the available data do not establish uninterrupted remission throughout the intervening period or the absence of additional treatment. The long-term finding therefore represents an association between initial treatment assignment and documented clinical status at the later assessment rather than proof that the initial four-week treatment produced continuous durable remission. The wide confidence intervals and sensitivity to assumptions regarding unavailable outcomes further reinforce the need for cautious interpretation.

The limited evidence base for conservative treatment consists predominantly of case reports, small series, and heterogeneous cohorts [1,11,12]. Published long-term observations also describe relapse after initial remission [27], supporting the importance of assessing outcomes beyond treatment completion without assuming that early improvement predicts lasting control. Other reported approaches include topical calcineurin inhibitors [25,28,29], combination preparations [30], mupirocin [31,32], imiquimod [33,34], carbon dioxide laser [35,36], and photodynamic therapy [37]. Differences in patient selection, treatment schedules, and outcome definitions preclude reliable indirect comparisons with the present cohort.

Our single-agent comparison does not evaluate combined corticosteroid–antibacterial treatment. Although fluticasone has an established role in dermatological practice [20,21] and fusidic acid monotherapy has previously been reported in plasma cell balanitis [22], neither pharmacological plausibility nor improvement in a small uncontrolled arm establishes an appropriate routine treatment strategy. The current findings do not support routine, repeated, or prolonged fusidic acid treatment for Zoon’s balanitis. Antimicrobial-stewardship considerations remain important [23,24], and the corrected guideline summary should not be read as an endorsement of this particular antibiotic regimen [19].

The low symptom burden is relevant to interpretation. Earlier cohorts also reported many minimally symptomatic or asymptomatic patients [3,10,25,30,36]. In our cohort, all symptom medians were zero at baseline, creating substantial floor effects. The pain imbalance was particularly marked: no participant in the fluticasone group reported baseline pain, compared with seven in the fusidic acid group. Consequently, the subsequent decrease in pain in the fusidic acid group cannot be interpreted as evidence of superiority over fluticasone. After Holm adjustment across the seven evaluable symptom-by-group longitudinal tests, only the reduction in pain in the fusidic acid group remained statistically significant. The symptom findings should therefore be regarded as exploratory rather than confirmatory.

Circumcision remains an important definitive-management option [3,10,19,38], but subsequent surgery should not automatically be interpreted as failure of the preceding topical treatment. Of the eight participants who underwent circumcision after the 3-month follow-up period, four underwent surgery because of worsening or recurrent Zoon’s balanitis, whereas four underwent circumcision according to patient preference rather than because of disease worsening; none underwent circumcision because of phimosis. Thus, the occurrence of circumcision reflects both disease-related and preference-related management decisions. In addition, incomplete information on intervening repeat or maintenance treatment limits attribution of the clinical status documented in 2026 solely to the initial treatment assignment. The condylomata in one fluticasone-treated participant responded to cryotherapy, but a single event without a fully characterised causal assessment does not establish drug causation or comparative safety. Absence of further recorded events must not be interpreted as evidence of long-term safety.

Strengths include prospective early assessments, histopathological confirmation as an inclusion requirement, repeated clinical assessment, and the inclusion of both physician-rated and patient-reported measures. However, several methodological limitations should be acknowledged. The study was conducted at a single centre with a relatively small sample size, and no formal a priori sample-size calculation was performed. Treatment allocation followed a predictable alternating sequence rather than concealed randomisation, and both treatment administration and clinical assessments were open-label, introducing potential selection and assessment biases. Unmeasured baseline factors may also have confounded the comparisons. Furthermore, treatment adherence was assessed during follow-up visits but was not systematically quantified. Differences in adherence between groups therefore cannot be excluded, particularly given the different application frequencies of fluticasone propionate (once daily) and fusidic acid (twice daily). The four-point IGA was adapted from a previously published clinical severity assessment but has not been formally validated specifically for Zoon’s balanitis, which may affect the reliability and comparability of the outcome measurements. In addition, outcome-specific missing or non-applicable entries required different denominators. The study was not prospectively registered, limiting the independent verification of prespecified outcomes and analyses. Moreover, the long-term assessment conducted in 2026 was not planned in the original study protocol and should therefore be considered exploratory. The available long-term data reflect categorical clinical status at a single later assessment rather than serial standardised measurements throughout the intervening period. Incomplete information on subsequent treatments and recurrence episodes prevents conclusions regarding uninterrupted treatment-free remission or the direct attribution of long-term outcomes to the initial treatment. Furthermore, four deaths and two losses to follow-up represent distinct forms of unavailable outcome data. Neither complete-case analysis nor extreme-assignment sensitivity analyses can fully eliminate the possibility of selection bias. The small sample size and limited systematic long-term safety monitoring also preclude reliable conclusions regarding the comparative safety of the two treatments, particularly with respect to uncommon adverse events or differences in their incidence. Therefore, the safety findings should be interpreted descriptively and with caution. Larger, prospectively registered studies with concealed random allocation, blinded outcome assessment where feasible, validated clinical outcome measures, prespecified handling of intercurrent events such as circumcision and death, and standardised longitudinal follow-up are needed to establish comparative efficacy and durability of treatment response.

## 5. Conclusions

Clinical severity improved during both four-week topical regimens, but the study did not establish comparative equivalence. Initial fluticasone allocation was associated with more frequent recorded remission at the exploratory 2026 assessment; this finding does not establish uninterrupted remission or a causal advantage in long-term durability. The small non-randomised cohort, incomplete follow-up and uncertain intervening management require cautious interpretation. The data do not justify routine or repeated fusidic acid treatment and support further registered, adequately powered comparative research.

## Figures and Tables

**Table 1:** Baseline demographics and clinical characteristics.

Characteristic	Fluticasone Propionate (*n* = 18)	Fusidic Acid (*n* = 19)	*p* Value *
Age, years	65.33 ± 14.74; 69 (29–80)	66.37 ± 10.87; 65 (43–84)	0.542
BMI, kg/m^2^	28.20 ± 3.17; 27.47 (21.95–34.68)	27.16 ± 3.35; 27.72 (23.24–35.26)	0.309
Symptom duration before diagnosis, months	21.39 ± 15.32; 21 (1–60)	26.47 ± 23.33; 24 (3–84)	0.678
Type 2 diabetes mellitus, *n* (%)	3 (16.7)	2 (10.5)	—
Immunosuppression, *n* (%)	0 (0.0)	0 (0.0)	—
Phimosis, *n* (%)	0 (0.0)	0 (0.0)	—
**Lesion location, *n* (%)**			
Glans only	5 (27.8)	3 (15.8)	
Foreskin only	1 (5.6)	2 (10.5)	
Glans and foreskin	12 (66.7)	14 (73.7)	
**Baseline IGA category, *n* (%)**			
IGA 1 (mild)	3 (16.7)	1 (5.3)	
IGA 2 (moderate)	9 (50.0)	12 (63.2)	
IGA 3 (severe)	6 (33.3)	6 (31.6)	
Current smoker, *n* (%)	0 (0.0)	1 (5.3)	—

* Between-group comparisons for continuous variables were performed using the Mann–Whitney U test. Data are presented as mean ± SD; median (range) for continuous variables and *n* (%) for categorical variables. BMI, body mass index; IGA, Investigator’s Global Assessment.

**Table 2:** Investigator’s Global Assessment (IGA) scores over time.

Time Point/IGA Category	Fluticasone Propionate (*n* = 18)	Fusidic Acid (*n* = 19)	*p* Value *
**Baseline**			
Mean ± SD; median (range)	2.17 ± 0.71; 2 (1–3)	2.26 ± 0.56; 2 (1–3)	0.718
IGA 0, *n* (%)	0 (0.0)	0 (0.0)	
IGA 1, *n* (%)	3 (16.7)	1 (5.3)	
IGA 2, *n* (%)	9 (50.0)	12 (63.2)	
IGA 3, *n* (%)	6 (33.3)	6 (31.6)	
**Week 2**			
Mean ± SD; median (range)	1.11 ± 0.58; 1 (0–2)	1.05 ± 0.71; 1 (0–3)	0.607
IGA 0, *n* (%)	2 (11.1)	3 (15.8)	
IGA 1, *n* (%)	12 (66.7)	13 (68.4)	
IGA 2, *n* (%)	4 (22.2)	2 (10.5)	
IGA 3, *n* (%)	0 (0.0)	1 (5.3)	
**Week 4 (end of treatment)**			
Mean ± SD; median (range)	0.67 ± 0.69; 1 (0–2)	0.89 ± 0.74; 1 (0–2)	0.338
IGA 0, *n* (%)	8 (44.4)	6 (31.6)	
IGA 1, *n* (%)	8 (44.4)	9 (47.4)	
IGA 2, *n* (%)	2 (11.1)	4 (21.1)	
IGA 3, *n* (%)	0 (0.0)	0 (0.0)	
**3 months after discontinuation**			
Mean ± SD; median (range)	1.00 ± 0.77; 1 (0–2)	1.11 ± 0.90; 1 (0–3)	0.762
IGA 0, *n* (%)	5 (27.8)	5 (26.3)	
IGA 1, *n* (%)	8 (44.4)	8 (42.1)	
IGA 2, *n* (%)	5 (27.8)	5 (26.3)	
IGA 3, *n* (%)	0 (0.0)	1 (5.3)	

* Between-group comparisons at each time point were performed using the Mann–Whitney U test. Within-group change over time (Friedman test): fluticasone propionate χ^2^(3) = 36.09, *p* < 0.001; fusidic acid χ^2^(3) = 32.93, *p* < 0.001. IGA was assessed on a 4-point scale (0–3), where 0 indicates no clinical signs and 3 indicates severe disease. Category counts are presented as *n* (%).

**Table 3:** Patient-reported symptom severity (VAS) over time.

Outcome/Time Point	Fluticasone Propionate (*n* = 18)	Fusidic Acid (*n* = 19)	*p* Value *
**Pain (VAS 0–10)**			
Baseline	0.00 ± 0.00; 0 (0–0)	1.63 ± 2.27; 0 (0–6)	0.005
Week 2	0.00 ± 0.00; 0 (0–0)	0.21 ± 0.92; 0 (0–4)	0.330
Week 4 (end of treatment)	0.00 ± 0.00; 0 (0–0)	0.21 ± 0.92; 0 (0–4)	0.330
3 months after discontinuation	0.00 ± 0.00; 0 (0–0)	0.16 ± 0.69; 0 (0–3)	0.330
**Burning (VAS 0–10)**			
Baseline	0.61 ± 1.38; 0 (0–5)	0.79 ± 1.90; 0 (0–6)	0.773
Week 2	0.17 ± 0.71; 0 (0–3)	0.21 ± 0.92; 0 (0–4)	1.000
Week 4 (end of treatment)	0.00 ± 0.00; 0 (0–0)	0.21 ± 0.92; 0 (0–4)	0.330
3 months after discontinuation	0.00 ± 0.00; 0 (0–0)	0.21 ± 0.92; 0 (0–4)	0.330
**Itch (VAS 0–10)**			
Baseline	0.67 ± 1.64; 0 (0–6)	1.16 ± 2.12; 0 (0–6)	0.460
Week 2	0.00 ± 0.00; 0 (0–0)	0.42 ± 1.12; 0 (0–4)	0.083
Week 4 (end of treatment)	0.00 ± 0.00; 0 (0–0)	0.32 ± 1.00; 0 (0–4)	0.163
3 months after discontinuation	0.00 ± 0.00; 0 (0–0)	0.21 ± 0.92; 0 (0–4)	0.330
**Dyspareunia (VAS 0–10)**			
Baseline	0.36 ± 1.34; 0 (0–5)	1.13 ± 2.09; 0 (0–6)	0.212
Week 2	0.00 ± 0.00; 0 (0–0)	0.38 ± 1.09; 0 (0–4)	0.178
Week 4 (end of treatment)	0.00 ± 0.00; 0 (0–0)	0.56 ± 1.55; 0 (0–5)	0.178
3 months after discontinuation	0.00 ± 0.00; 0 (0–0)	0.38 ± 1.09; 0 (0–4)	0.178

* Between-group comparisons at each time point were performed using two-sided Mann–Whitney U tests. For pain, burning, and itch, denominators were *n* = 18 for fluticasone propionate and *n* = 19 for fusidic acid. Dyspareunia was evaluated only in participants with an evaluable numeric response (*n* = 14 and *n* = 16, respectively); participants with documented absence of sexual activity were classified as not applicable and excluded from this analysis. Data are presented as mean ± SD; median (range). VAS, visual analogue scale.

**Table 4:** Exploratory Long-Term Clinical Outcomes Assessed in 2026.

Outcome	Fluticasone Propionate (*n* = 18)	Fusidic Acid (*n* = 19)	Overall (*n* = 37)
Recorded remission at the 2026 assessment	12 (66.7%)	3 (15.8%)	15 (40.5%)
Persistent/recurrent disease without remission	2 (11.1%)	6 (31.6%)	8 (21.6%)
Circumcision after topical treatment	2 (11.1%)	6 (31.6%)	8 (21.6%)
Not assessable: lost to follow-up or deceased	2 (11.1%)	4 (21.1%)	6 (16.2%)
Among assessable patients only			
Assessable patients	16 (88.9%)	15 (78.9%)	31 (83.8%)
Recorded remission at the 2026 assessment	12/16 (75.0%)	3/15 (20.0%)	15/31 (48.4%)
Persistent/recurrent disease without remission	2/16 (12.5%)	6/15 (40.0%)	8/31 (25.8%)
Circumcision after topical treatment	2/16 (12.5%)	6/15 (40.0%)	8/31 (25.8%)

Long-term outcome categories were mutually exclusive. Circumcision was performed after completion of the 3-month follow-up period. Of the eight circumcisions, four were performed because of worsening or recurrent Zoon’s balanitis and four according to patient preference rather than because of disease worsening.

## Data Availability

The de-identified data underlying this article are available from the corresponding author upon reasonable request, subject to institutional and ethical restrictions.

## Supplementary Materials

The following supporting information can be downloaded at https://www.mdpi.com/article/10.3390/jcm15218515/s1, Table S1: Exploratory Categorical Early Responses; Table S2: Sensitivity analyses for unavailable 2026 outcomes.

## Abbreviations

The following abbreviations are used in this manuscript:

IGA	Investigator’s Global Assessment
VAS	Visual Analogue Scale
ZB	Zoon’s balanitis
NFKB1	Nuclear factor kappa B subunit 1
NFKB2	Nuclear factor kappa B subunit 2
mRNA	Messenger RNA
