# Supplementary material for: Topical Fluticasone Propionate Versus Fusidic Acid for Zoon’s Balanitis in Men: A Prospective, Open-Label, Non-Randomised Comparative Study with an Exploratory Long-Term Assessment
Source: J Clin Med. 2026 Nov 2;15(21):8515. doi: 10.3390/jcm15218515 (PMC13764407; doi:10.3390/jcm15218515)
Supplement: Supplementary file 1 [file jcm-15-08515-s001.zip › jcm-4566499-supplementary.pdf]

Table S1. Exploratory Categorical Early Responses

Visit	Outcome	Fluticasone	Fusidic acid	Difference, percentage points (95% CI)	Fisher p
Week 4	Complete clearance (IGA 0)	8/18 (44.4%)	6/19 (31.6%)	12.9 (-17.1 to 40.1)	0.50768
Week 4	Clear or mild disease (IGA 0–1)	16/18 (88.9%)	15/19 (78.9%)	9.9 (-15.1 to 33.6)	0.65988
Week 4	Improvement ≥1 grade	16/18 (88.9%)	16/19 (84.2%)	4.7 (-19.3 to 27.9)	1.00000
3 months	Complete clearance (IGA 0)	5/18 (27.8%)	5/19 (26.3%)	1.5 (-25.7 to 28.7)	1.00000
3 months	Clear or mild disease (IGA 0–1)	13/18 (72.2%)	13/19 (68.4%)	3.8 (−24.4 to 30.9)	1.00000
3 months	Improvement ≥1 grade	14/18 (77.8%)	13/19 (68.4%)	9.4 (-18.8 to 35.4)	0.71398

* Data are presented as n/N (%). Differences are fluticasone minus fusidic acid and are expressed in percentage points with 95% confidence intervals calculated using Newcombe's hybrid-score method. Between-group comparisons were performed using two-sided Fisher's exact tests.
These categorical response analyses were exploratory. IGA, Investigator's Global Assessment.

Table S2. Sensitivity analyses for unavailable 2026 outcomes.

Analysis	Fluticasone	Fusidic acid	Difference, percentage points (95% CI)	Fisher p
Assessable participants	12/16 (75.0%)	3/15 (20.0%)	55.0 (19.9 to 74.7)	0.00385
Full cohort: documented remission	12/18 (66.7%)	3/19 (15.8%)	50.9 (19.3 to 70.8)	0.00255
All six unassessable outcomes: adverse mathematical bound for fluticasone	12/18 (66.7%)	7/19 (36.8%)	29.8 (-2.0 to 54.4)	0.10314
All six unassessable outcomes: favourable mathematical bound for fluticasone	14/18 (77.8%)	3/19 (15.8%)	62.0 (30.3 to 78.7)	0.00023
Deaths excluded; both living losses assigned no remission	12/17 (70.6%)	3/16 (18.8%)	51.8 (17.9 to 72.0)	0.00490
Deaths excluded; both living losses assigned remission	13/17 (76.5%)	4/16 (25.0%)	51.5 (17.4 to 71.8)	0.00527
Deaths excluded; favourable living-loss assignment for fluticasone	13/17 (76.5%)	3/16 (18.8%)	57.7 (23.8 to 76.2)	0.00155

Deaths excluded; adverse living-loss assignment for fluticasone	12/17 (70.6%)	4/16 (25.0%)	45.6 (11.5 to 67.5)	0.01492

* Nominal, two-sided Fisher p values. All-six extreme assignments are mathematical bounds and do not impute that deceased participants were alive and in remission in 2026. Four participants were deceased (F: 1; A: 3), and two others were lost to follow-up (F: 1; A: 1). Analyses excluding deaths describe a selected survivor population. Missingness assumptions do not remove confounding or prove causal durability.
